# Professor Karin Diserens, WFNR chair of the Special Interest Group on Early Mobilization and member of the executive committee of the EFNR Health & Economics: Adapted Interview from the 12^th^ World Congress for Neurorehabilitation (WCNR), Vienna, 2022

**DOI:** 10.25122/jml-2023-1019

**Published:** 2023-04

**Authors:** Stefana-Andrada Dobran, Alexandra Gherman

**Affiliations:** 1 RoNeuro Institute for Neurological Research and Diagnostic, Cluj-Napoca, Romania; 2 Sociology Department, Babes-Bolyai University, Cluj-Napoca, Romania

**Interviewer: Stefana-Andrada Dobran**

**Interviewee: Professor Karin Diserens °**

° Acute Neurorehabilitation Unit

Service of Neurology and Department of Clinical Neurosciences, University Hospital of Lausanne, Switzerland

Professor Karin Diserens is a specialist in neurology and physical medicine and rehabilitation. She co-founded the Swiss Society for Neurorehabilitation and currently serves as the President of the Swiss Neurobehavior Society. Previously, she was the head of the post-acute neuro-rehabilitation clinic (1996-2005) and now leads a mobile transversal neurorehabilitation team at the Lausanne University Hospital (2006-2009) as head of the Acute Neuro-rehabilitation Unit of the Neurology service (NRA), Department of Clinical Neurosciences, Lausanne University Hospital.

Professor Diserens has made significant contributions to the evaluation of quality criteria for acute and post-acute neurorehabilitation in Switzerland, and her current research focuses on the diagnosis of disorders of consciousness and the effect of interdisciplinary neurosensorial stimulation and hyper-acute mobilization using robotic mobilization to facilitate recovery in patients with severe neurological and traumatic lesions. The NRA Unit places great emphasis on a cognitive approach to creation and emotion and develops innovative treatment techniques for indoor and outdoor therapy. As a university lecturer (Privat docent), she created a teaching program for acute neurorehabilitation for pre-graduate and post-graduate training of medical students and interdisciplinary professionals. Karin Diserens was nominated Professor at Lausanne University, Switzerland, in 2021.

**S.A.D.:** Dear Professor Karin Diserens, we are here in Vienna for the 12^th^ World Congress for Neurorehabilitation, organised by the WFNR. What is your first-hand opinion of the event so far, and have you participated in any previous editions?

**K.D.:** Yes, I have participated regularly; I am the co-chair of the SIG (Special Interest Group) on Rehabilitation, and, in this function, I participated in the scientific program. The impression I have is that with each congress the WFNR manages to bring together specialists in many different domains; it is a very dynamic platform to exchange in-between medical and therapeutic specialists.

**S.A.D.:** And how was the first congress you participated in, in Istanbul?

**K.D.:** Yes, the first congress was in Istanbul. It was very interesting because right from the start, we had very good participation from several countries and many different specialties. This collaboration has continued in all the years after.

**S.A.D.:** What do you believe is the overarching theme of this year’s congress?

**K.D.:** Each congress attends to the main subject of rehabilitation. This year, we managed to integrate innovative techniques for diagnosis, prognostication, and treatment of disorders of consciousness.

**S.A.D.:** How do you believe that interdisciplinary interaction plays a role in this event?

**K.D.:** Interdisciplinary interaction is crucial when dealing with complex disorders, bringing together a mixture of views and ideas to form new perspectives.

**S.A.D.:** What are some limitations regarding access to care for patients suffering from disorders of consciousness?

**K.D.:** Disorders of consciousness are a very controversial subject area, especially today in spite of the progress in neurophysiology and neuroradiology. One of the main limitations is that the definition of the type of disorder of consciousness is not the same in every country (and even within countries) and the methodology to evaluate disorders of consciousness is not the same either.

**S.A.D.:** How is the diagnosis during the acute phase in these disorders?

**K.D.:** The diagnosis in the acute phase is very important, and it should not be limited to clinical scores. The focus of our research, especially in our group, is to enlarge the clinical evaluation by observation of motor behaviour and complemented by a simultaneous multimodal assessment.

**S.A.D.:** Alright. What are the prerequisites for an integrative approach to disorders of consciousness?

**K.D.:** First of all, we need to choose an adequate methodology to reach a diagnosis as precisely as possible; as I said, standardized scales should accommodate the assessment of motor behaviour. We have to look at the individual goals of the patient and their family, already in the acute phase when sedation is stopped. There should be clear communication between the professionals involved in the patient management pathway, including neurologists, neurosurgeons, intensivists, and continuing with the therapeutic teams for the post-acute phase and especially for the later ambulatory phase. The initial goal assessment in the acute phase is therefore very important.

**S.A.D.:** What are the main advantages and limitations of brain-computer interfaces (BCI) and Virtual Reality developments in contrast to classical rehabilitation?

**K.D.:** The advantages of brain-computer interface are that patients who are unable to communicate, like locked-in patients or who lack mobility, are provided the ability to interact with their environment via their brain activity; nevertheless, we have to be very careful with patients who cannot interact or command robot simulations: do they have a physical or vigilance problem, can they give a robust signal on EEG? I.e., we must choose appropriate patients for BCI. In addition, the BCI task needs to be motivating for the patient.

**S.A.D.:** And who can decide when the patient is autonomous?

**K.D.:** With **BCI**, we can give the patient a larger autonomy, but it may be very difficult for the patient to do this and perhaps too difficult to concentrate, so we should look for other possibilities to interact with the patient. The same is true for Virtual Reality approaches; it is a new innovative technique, very interesting, very motivating, but the patient must be cognitively fit to follow the virtual reality paradigm and to be able to use it. If the patient can use it well, an advantage of VR is the possibility to do this stimulating rehabilitation as ambulatory follow-up treatment.

**S.A.D.:** My last question would be, are the outcomes of disorders of consciousness sufficiently evaluated in the short-term/long-term?

**K.D.:** This is still being discussed and researched. We must choose appropriate evaluation scales, and must also determine an individual goal assessment, tailored to the patient and based on the International Classification of Functioning, Disability and Health (ICF), which is an excellent approach that guides us to be in line with the patient’s goals, family’s goals, and specialists’ goals.

**S.A.D.:** Thank you for the interview!

**K.D.:** Thank you!

